# Regulatory Feedback Loop of Two *phz* Gene Clusters through 5′-Untranslated Regions in *Pseudomonas* sp. M18

**DOI:** 10.1371/journal.pone.0019413

**Published:** 2011-04-29

**Authors:** Yaqian Li, Xilin Du, Zhi John Lu, Daqiang Wu, Yilei Zhao, Bin Ren, Jiaofang Huang, Xianqing Huang, Yuhong Xu, Yuquan Xu

**Affiliations:** 1 State Key Laboratory of Microbial Metabolism, School of Life Sciences and Biotechnology, Shanghai Jiao Tong University, Shanghai, China; 2 Department of Molecular Biophysics and Biochemistry, Yale University, New Haven, Connecticut, United States of America; 3 School of Pharmacy, Shanghai Jiao Tong University, Shanghai, China; Queen Mary University of London, United Kingdom

## Abstract

**Background:**

Phenazines are important compounds produced by pseudomonads and other bacteria. Two *phz* gene clusters called *phzA1-G1* and *phzA2-G2*, respectively, were found in the genome of *Pseudomonas* sp. M18, an effective biocontrol agent, which is highly homologous to the opportunistic human pathogen *P. aeruginosa* PAO1, however little is known about the correlation between the expressions of two *phz* gene clusters.

**Methodology/Principal Findings:**

Two chromosomal insertion inactivated mutants for the two gene clusters were constructed respectively and the correlation between the expressions of two *phz* gene clusters was investigated in strain M18. Phenazine-1-carboxylic acid (PCA) molecules produced from *phzA2-G2* gene cluster are able to auto-regulate expression itself and activate the expression of *phzA1-G1* gene cluster in a circulated amplification pattern. However, the post-transcriptional expression of *phzA1-G1* transcript was blocked principally through 5′-untranslated region (UTR). In contrast, the *phzA2-G2* gene cluster was transcribed to a lesser extent and translated efficiently and was negatively regulated by the GacA signal transduction pathway, mainly at a post-transcriptional level.

**Conclusions/Significance:**

A **single** molecule, PCA, produced in **different** quantities by the two *phz* gene clusters acted as the functional mediator and the two *phz* gene clusters developed a specific regulatory mechanism which acts through 5′-UTR to transfer a single, but complex bacterial signaling event in *Pseudomonas* sp. strain M18.

## Introduction

Phenazines are important heterocyclic nitrogen-containing compounds produced by some strains of fluorescent *Pseudomonas* spp. and a few other bacterial genera. The biological functions of phenazines include roles both as signaling molecules and antibiotics, ultimately contributing to the microbe's behavior and ecological suitability for survival in a competitive environment [1]–[3]. The extensive diversity and ecological fitness experienced during microbial evolution was confirmed by studies on the distribution of phenazine genes in different phenazine-producing bacteria originating from various environmental niches [4], [5]. The core phenazine biosynthetic gene cluster, *phz*, has been shown to move among diverse bacterial genera via horizontal gene transfer, thereby driving genetic diversity in phenazine-producing bacteria [6]. Over the past decade, significant progress has been made toward our understanding of the genetic, signaling regulation, and functional roles of various phenazines, with a particular focus having been placed on the pseudomonads [7]–[25].

Though the core *phz* operon responsible for phenazine-1-carboxylic acid (PCA), a precursor of phenazines, biosynthesis is highly conserved among various pseudomonads [6], [10], the pseudomonads are divided into two categories based on the copy numbers of the core *phz* gene cluster contained within the chromosomal genome. The first category is composed of *Pseudomonas* spp. containing only one set of the *phz* gene cluster, and includes strains of *P. fluorescence* 2–79, *P. aureofaciens* 30–84 and *P. chlororaphis* PCL1391 and other strains [12]–[14]. These particular pseudomonads are known as plant growth promoting rhizobacteria (PGPRs), and inhibit soil-born phytopathogenic fungi and are beneficial to plant growth and crop production [15], [16]. Moreover, the PGPRs have evolved a high reliance on plant -specific environment and identification of cross-species infection in animal or human has been extremely rare. However, two nearly identical core *phz* gene clusters, called *phzA1-G1* and *phzA2-G2*, but with different promoters and flanking regions, have been found in the second category of pseudomonads, which include the most extensively studied phenazine-producer strain *P. aeruginosa* PAO1 [17]. *P. aeruginosa* is a well-characterized opportunistic pathogen of animal and human, and is ubiquitously distributed throughout soil and aquatic habitats [18]–[20]. The location of two *phz* gene clusters and their flanking genes have been well characterized in various *P. aeruginosa* genomes. In particular, the functions of the *phzM* and *phzS* genes flanking *phzA1-G1* are known to play a critical role in the conversion of PCA into pyocyanin (PYO) [17], [21]–[22], [32]. PYO is considered as the predominant phenazine produced from all the nosocomial originating *P. aeruginosa* strains at 37°C [23]. Furthermore, PAO1 strain genome analyses have revealed a gene, *qscR*, that is similar to *lasR* and *rhlR* of the quorum sensing (QS) system and is located upstream of *phzA2-G2* [24], [25]. However, the regulatory mechanism involved in correlation between the expressions of two *phz* gene clusters is poorly understood.

*Pseudomonas* sp. M18, isolated from the rhizosphere of a sweet-melon in Shanghai suburb in China has been used as an effective biological control strain against various soil-born phytopathogens [26] and shares a similar genetic background with the opportunistic human pathogen *P. aeruginosa* PAO1. The 16S rRNA gene sequence of strain M18 (AY696302) is 99% identical to PAO1, and several regulatory genes such as *gacA*, *gacS*, *rsmA*, *rpoS*, *qscR*, *vqsR*, *lasIR* and *rhlIR* share more than 90% sequence similarity with those in *P. aeruginosa* PAO1 [27]–[30]. Though *Pseudomonas* sp. M18 seems to share a common ancestor with *P. aeruginosa* PAO1, it has developed distinctive features consistent with its rhizosphere niche. First, both PCA and the chlorinated antibiotic pyoluteorin can be produced in a single M18 strain, resulting in strong synergistic activities to protect plants against various fungal phytopathogen infections. Second, expression of the phenazine-decorating gene *phzM* responsible for the conversion of PCA into PYO is temperature dependent at 37°C; the predominant phenazine produced by strain M18 at 28°C is PCA, rather than PYO [31], as PYO is not essential for fungi killing [32]. Third, phenazine production is negatively regulated by the global regulator GacA in *Pseudomonas* sp. M18, especially at 28°C [27]; in contrast, it is positively regulated in *P. aeruginosa* PAO1 [33].

In the study presented herein, we aim to elucidate the correlation between the expressions of two *phz* gene clusters and to specify the negative regulation mechanism of GacA signal transduction system on PCA synthesis in *Pseudomonas* sp. M18. This work represents the first description of a regulatory feedback loop involving the expressions of two *phz* gene clusters through non-coding regions and the adoption of a two-*phz*-gene-cluster cascade mechanism to transfer a single, but complex, bacterial signaling event in *Pseudomonas* sp. strain M18.

## Results

**Figure 1:**
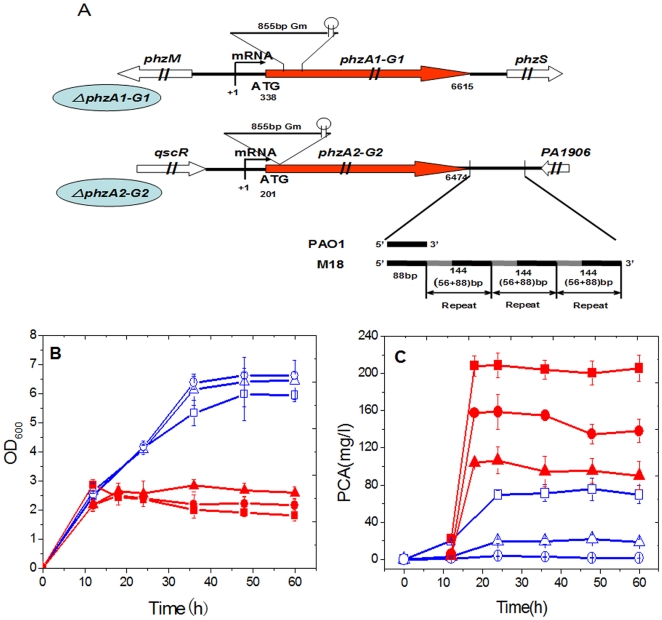
The physical map, growth and PCA production of strain M18 and its two *phz* mutants. The two inactivated *phz* gene clusters with their flanking genes in *Pseudomonas* sp. M18 (A) and the growth curves (B) and PCA production (C) in wild type strain and the two mutants M18ΔP1 and M18ΔP2 with or without exogenous PCA molecules. Symbols in figure 1A: red solid arrows denote the two *phz* core gene clusters; empty arrows, the *phz* flanking genes; fine line, Gm, genamycin resistence gene; +1, indicates the transcriptional start site (TSS) and the numbers indicate the relative length from TSS; black lines, intergenic regions between *phz* gene clusters and flanking genes; black solid lines denote the 88 bp sequence homologous to that in *P. aeruginosa* PAO1; grey solid line denotes additional 56 bp sequence in strain M18. Symbols in figure 1B and 1C: square, wild type strain M18; triangle, mutant M18ΔP1; circle, mutant M18ΔP2; red solid and blue empty denote presence or absence of exogenous PCA in culture, respectively. All experiments were performed in triplicate, and each value is presented as the average ± standard deviation.

In our previously published paper [34], the two *phz* gene clusters along with their flanking sequences in *Pseudomonas* sp. M18 genome were sequenced and deposited into GenBank with accession numbers FJ494908 and FJ494909. The alignment of nucleotide sequences revealed that the two gene clusters share 99% homology with sequences in *P. aeruginosa* PAO1, each consisting of seven coding genes, *phzA1B1C1D1E1F1G1* (*phzA1-G1*) and *phzA2B2C2D2E2F2G2* (*phzA2-G2*), as shown in Figure 1A. The flanking genes of the two clusters are also similar to that found in *P. aeruginosa* PAO1, except for a 520 bp interval gene region directly downstream the *phzA2-G2* gene cluster between the *phzG2* gene and an ORF PA1906; instead, a short 88 bp sequence is present in strain PAO1. This finding indicated that the genetic background of strain M18 differed from that of strain PAO1, though the function of the repeat region remains unknown.

### A positive feedback loop of PCA produced from the two *phz* gene clusters

Two chromosomal insertion inactivated mutants for the *phzA1-G1* and *phzA2-G2* gene clusters were constructed respectively in *Pseudomonas* sp. M18 and designated as M18ΔP1 and M18ΔP2 (Figure 1A). We first investigated the relationship between the expressions and regulation of two *phz* gene clusters by quantification analysis of cell growth and PCA production in the wild type strain M18 and the two mutant strains as shown in Figure 1B & 1C. The results indicated that M18ΔP1 and M18ΔP2 mutants experienced slight increases in the cell growth, as compared with the wild type strain M18. During the stationary phase, the cell growth of the *phzA2-G2* inactivated mutant M18ΔP2 was the fastest among the three strains tested, while the *phzA1-G1* mutant M18ΔP1 exhibited more rapid growth than the wild type strain M18, suggesting the possibility of a toxic inhibition effect on the cell growth created by certain PCA concentrations and the different amounts of PCA produced in these strains (Figure 1B). The PCA toxic effect on cell growth was confirmed when we quantified PCA production from the three strains, as shown in Figure 1C. It was found that PCA production from wild type strain M18 was almost 4-fold higher than that from the *phzA1-G1* inactivated mutant M18ΔP1 and PCA production from the *phzA2-G2* inactivated mutant M18ΔP2 was abolished completely, suggesting that PCA could not be produced from the gene cluster *phzA1-G1* in the absence of PCA molecules produced from the *phzA2-G2* gene cluster. Specifically, 20 mg/L PCA was found to be produced by the mutant M18ΔP1, which was only one-quarter of the PCA production detected from the strain M18, suggesting that the gene cluster *phzA2-G2* could only produce a small amount of PCA and that the majority of PCA molecules were produced from the *phzA1-G1* gene cluster in the strain M18. These results suggested that PCA molecules produced from the *phzA2-G2* gene cluster could activate the expression of *phzA1-G1* gene cluster to produce more PCA molecules in *Pseudomonas* sp. M18. We deduced that the expressions of the two *phz* gene clusters may represent a feedback amplification pattern.

Therefore, experiments were further carried out using the addition of exogenous PCA into cultures to confirm the above findings. As shown in Figure 1B, supplementing the cultures with exogenous PCA resulted in significantly inhibited growth of the three strains, especially for the wild type strain during the growth phase, indicating a toxic effect of PCA on cell growth. On the other hand, the addition of exogenous PCA could induce the two mutant strains to produce more PCA and stimulated the wild type strain M18 to produce a large amount PCA of 220 mg/L (Figure 1C). Taken together, our results suggested that there exists a PCA auto-induction regulatory mechanism involving expression of the *phzA2-G2* gene cluster and an activation mechanism involving expression of the *phzA1-G1* gene cluster. The increase of PCA production with exogenous PCA at 160 mg/L in M18ΔP2 and 70 mg/L in M18ΔP1 indicated a higher efficiency induction for *phzA1-G1* cluster and a relatively lower efficiency induction for *phzA2-G2* cluster, which again supported the function of a positive feedback loop involved in PCA production in *Pseudomonas* sp. M18 strain.

### Transcription of the *phzA1-G1* gene cluster, rather than *phzA2-G2*, was efficiently activated by PCA molecules

The activating function of PCA as a signaling molecule was examined in regard to its effect on which one transcriptional activity of the two *phz* gene clusters Total *phz* transcriptional level was measured and compared to the levels of *phzA1-G1* and *phzA2-G2*, respectively, in the presence or absence of exogenous PCA by using quantitative real time PCR (QRT-PCR) method. As shown in Table 1, the relative *phz* gene expression levels obtained from the three strains without addition of exogenous PCA were much lower than those obtained with exogenous PCA addition, which verified the transcriptional induction function of PCA molecules for the expressions of the two *phz* gene clusters. Notably, the relative expression activities of *phzA1-G1* and *phzA2-G2* increased by 18.4- and 5.7- fold when the two M18ΔP2 and M18ΔP1 mutants were incubated in the presence of exogenous PCA, indicating a higher transcriptional potential of the *phzA1-G1* gene cluster and a more robust transcriptional response for this gene cluster to the PCA molecules, when compared with that of *phzA2-G2* cluster. Compared with the M18ΔP1 mutant, the *phzA2-G2* transcript level could be induced to a much less extent with the addition of exogenous PCA molecules, suggesting the lower transcriptional potential of the *phA2-G2* gene cluster and less robust auto-induction efficiency of this cluster. It could be deduced that the PCA produced from the *phzA2-G2* cluster plays a signaling role to induce the expression of itself in a relatively less efficient manner, however to activate the expression of *phzA1-G1* gene cluster more efficiently.

**Table 1:** Effects of exogenous PCA on the transcription of two *phz* gene clusters in *Pseudomonas* sp. M18 and its two mutants M18ΔP1 and M18ΔP2.

Strains	Without exogenous PCA				With exogenous PCA				Fold
	CT*rpoD* a	CT*phz* a	ΔCT^c^	Z1^d^	CT*rpoD* b	CT*phz* b	ΔCT^c^	Z2^d^	M^e^
M18	16.6±0.1	22.8±0.2	6.2	2.8	17.0±0.5	19.8±0.3	2.8	5.3	10.6
M18ΔP1	16.9±0.3	24.6±0.2	7.7	1.0	16.3±0.3	22.5±0.2	5.2	1.0	5.7
M18ΔP2	17.1±0.1	24.2±0.1	7.1	1.5	16.6±0.2	19.5±0.1	2.9	4.9	18.4

a Values were measured during exponential phase at an OD_600_ of 2.0 to 2.5. CT, cycle threshold.

b Values were measured at stationary phase at OD_600_ of 3.5 to 4.0.

c ΔCT = CT*phz*−CT*rpod*, CT*phz* denotes cycle threshold of *phzC*, *phzA2* and *phzA1* transcripts in strain M18, M18ΔP1 and M18ΔP2, respectively.

d Z1 or Z2 = 2^−Δ(ΔCT*phz*C-ΔCT*phzA2*)^ (in M18); 2^−Δ(ΔCT*phzA1*-ΔCT*phzA2*)^ (in M18ΔP2); 2^−Δ(ΔCT*phzA1*-ΔCT*phzA2*)^ (in M18ΔP1), ΔΔCT = ΔCT_low_−ΔCT_high_ (The relative high ΔCT value was defined as 1).

e M, the increased ratio of expression 2^−ΔΔCT^ without and with the addition of exogenous PCA in culture. ΔΔCT = ΔCT_with_–ΔCT_without_.

### The efficiently activated expression of *phzA1-G1* gene cluster was blocked at a post-transcriptional level

**Figure 2:**
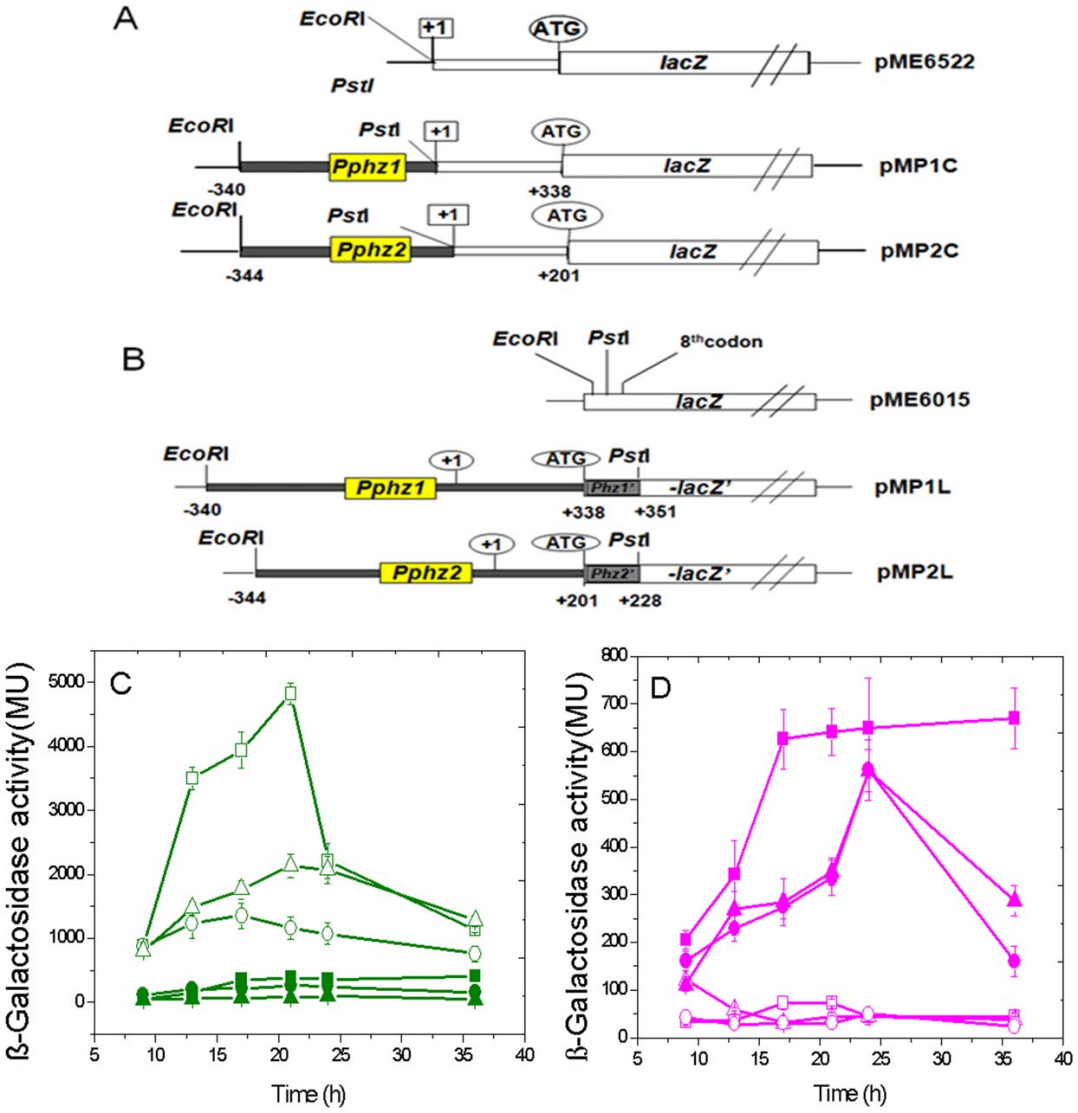
Activities of two *phz‘*-*’lacZ* transcriptional or translational fusions in strain M18 and its two mutants. The *phz1‘-’lacZ* and *phz2‘-’lacZ* transcriptional or translational fusion in plasmids pME6522 (A) or pME6015 (B). Their transcriptional (C) or translational activities (D) in wild type strain *Pseudomonas* sp. M18 (square), *phzA1-G1* inactivated mutant M18ΔP1 (triangle) and *phzA2-G2* inactivated mutant M18ΔP2 (circle). Symbols: open, *phz1‘-’lacZ* fusion; solid, *phz2‘-’lacZ* fusion. All experiments were performed in triplicate, and each value is presented as the average ± standard deviation.

The different transcription activity and induction efficiency of the two *phz* gene clusters by PCA molecules instigated us to make a further investigation on the regulation of two gene clusters at a post-transcriptional level. The transcriptional start sites of each gene cluster, *phzA1-G1* and *phzA2-G2*, were determined by a rapid amplification of 5′-cDNA element (5′-RACE) method and a long non-coding region of 338 bp and 201 bp was found between the transcriptional start point and the predicted translation start point in the two gene clusters, respectively (Figure 1A). Two transcriptional fusions, *phz1‘*-*’lacZ* and *phz2‘*-*’lacZ*, were constructed in pME6522, and called as pMP1C and pMP2C (Figure 2A). The β-Galactosidase activities from the two transcriptional fusions were measured both in wild type strain M18 and the two M18ΔP1 and M18ΔP2 mutants, respectively (Figure 2C). It was evident that β-Galactosidase activity from the *phz1‘*-*’lacZ* transcriptional fusion was much higher than that from the *phz2‘*-*’lacZ* whether in the wild type strain M18 or in the two *phz* mutants. Also, the *phz1‘*-*’lacZ* transcriptional activity was higher in the M18ΔP1 mutant than that in M18ΔP2, though much less than that in wild type strain M18. In contrast, the *phz2‘*-*’lacZ* transcriptional fusion expressed at a relatively lower level and did not show significant differences among the three strains. These data indicated that the *phzA2-G2* gene cluster was transcribed less efficiently than *phzA1-G1* gene cluster and the endogenous PCA produced from the *phzA2-G2* gene cluster could induce the expression of *phzA1-G1* gene cluster more efficiently at the transcriptional level. Moreover, *phzA1-G1* gene cluster could be induced and expressed by some other unknown factor(s) in the M18ΔP2 mutant in the absence of endogenous PCA molecules.

The results were consistent with findings obtained from quantitative *phz* transcript analysis by QRT-PCR, but were not consistent with the PCA quantity produced in wild type strain M18 or in the two mutants, suggesting a repression mechanism involved in the *phzA1-G1* transcript and not in the *phzA2-G2* transcript exists at the post-transcriptional levels in strain M18. The two translational fusions of *phz1‘*-*’lacZ* and *phz2‘*-*’lacZ* named as pMP1L and pMP2L were further constructed in pME6015 (Figure 2B) and introduced into wild type strain M18 and the two *phz* mutants of M18ΔP1 and M18ΔP2, respectively. The β-Galactosidase activities from the two translational fusions were measured in the three strains, respectively, as shown in Figure 2D. As expected, the β-Galactosidase activity from pMP2L translational fusion was much higher in comparison with that from pMP1L translational fusion either in the wild type strain or in the M18ΔP1 and M18ΔP2 mutants, indicating that the *phzA2-G2* transcript could be expressed efficiently at the translational level and activated less efficiently by endogenous PCA produced from *phz* gene cluster or the unknown factor(s) besides PCA molecules. However, the β-Galactosidase activity from translational fusion pMP1L was nearly completely blocked in the three strains, indicating that the efficiently transcribed *phzA1-G1* transcript was poorly translated, even under the conditions of endogenous PCA induction in the wild type strain M18 and the mutant M18ΔP1. This finding suggested that there exists a powerful negative *cis* control mechanism located in the non-coding region of *phzA1-G1*, rather than in the *phzA2-G2* transcripts, which could act as a repressor to block the translation from the *phzA1-G1* transcripts.

### The *phzA1-G1* post-transcriptional event was blocked by two domains located in the 5′-untranslated region

**Figure 3:**
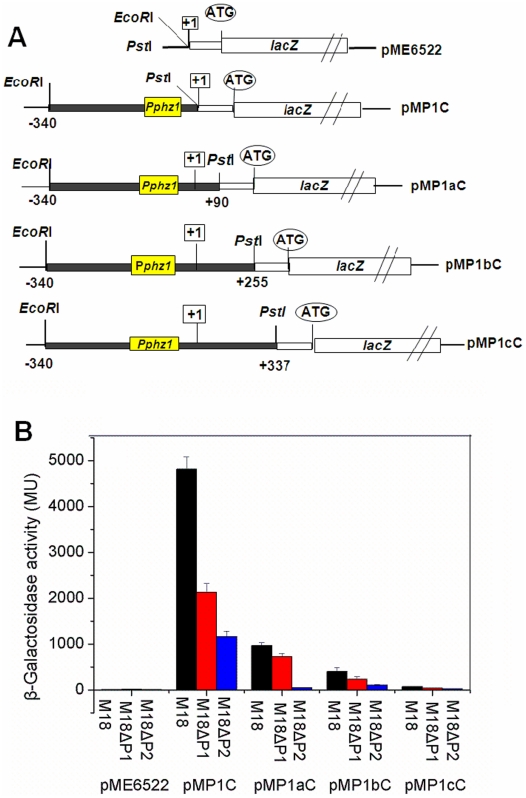
Activities of various *phz1‘-’ lacZ* transcriptional fusions during logarithmic phase in strain M18 and its mutants. The various *phz1‘-’lacZ* transcriptional fusions in plasmid pME6522 (A) and their β-Galactosidase activities (B) in wild type strain *Pseudomonas* sp. M18 (black), *phzA1-G1* inactivated mutant M18ΔP1 (red) and *phzA2-G2* inactivated mutant M18ΔP2 (blue). All experiments were performed in triplicate, and each value is presented as the average ± standard deviation.

The above results forced us to construct another three transcriptional fusions, pMP1aC pMP1bC and pMP1cC in pME6522 (Figure 3A), and to investigate the transcriptional activities of these fusions in the wild type and the two *phz* mutants. As shown in Figure 3B, when a region of 90 bp in length was recovered into the 5′-untranslated region (UTR), the β-Galactosidase activity resulting from the transcriptional fusion pMP1aC was 5-fold less than that from the transcription fusion pMP1C in the wild type strain. When a region of 337 bp in length was recovered into the 5′-UTR, the β-Galactosidase activity from pMP1cC fusion was 6-fold less than that from pMP1bC in the wild type M18, indicating that two negative control elements from +1 to +90 nt and from +255 to +337 nt were contained in the 5′-UTR of the *phzA1-G1* transcript. The same trend was found for the expressions of all the transcriptional fusions in M18ΔP1 and M18ΔP2; however, when compared to that in the wild type strain, only about half of the β-Galactosidase activity from the transcription fusion pMP1C was detected in the M18ΔP1 and one-quarter in M18ΔP2, suggesting that some other unknown factor(s) in the mutants, besides PCA molecules, may be involved in the post-transcriptional or translational regulation of the *phzA1-G1* transcript. The β-Galactosidase activities of pMP1aC and pMP1cC in strain M18ΔP1 increased 14.4-fold and 1.5-fold, respectively, compared with that in strain M18ΔP2, suggesting that PCA produced from the *phzA2-G2* cluster was able to induce *phzA1-G1* expression mainly through a mechanism involving the 5′-UTR regions from +1 to +90 nt; although the precise mechanism(s) by which the two *cis*-element domains regulate the *phzA1-G1* expression flexibly at the post-transcriptional level remains unknown.

**Figure 4:**
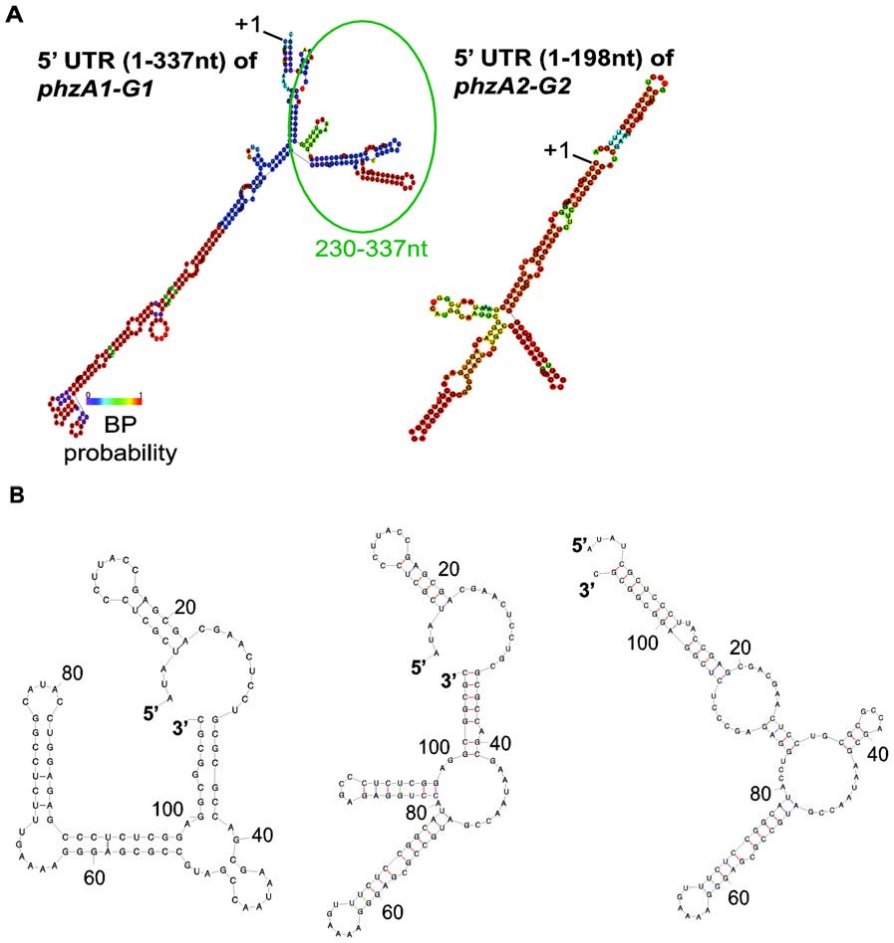
Secondary structures in the 5′-UTRs of two *phz* gene clusters were predicted by RNA fold. The predicted secondary structures in 5′-UTR of *phzA1-G1* (1–337 nt) and *phzA2-G2* (1–198 nt) gene clusters (A). The conserved RNA secondary structures were predicted from the alignment of *Pseudomonas* sp. M18 and *P. aeruginosa* PA7. The base pairing probabilities were annotated with colors. Three suboptimal secondary structures were also predicted for a portion of the 5′-UTR of the *phzA1-G1* gene cluster in *Pseudomonas* sp. M18 (B). Gibbs free energies (ΔG) of the three suboptimal local structures from left to right are −39.3 kcal/mol, −38.1 kcal/mol and −36.7 kcal/mol, respectively.

We predicted the secondary structures of the 5′-UTR from the *phzA1-G1* gene cluster (Figure 4A). In order to find the conserved secondary structures with more accuracy, the 5′-UTRs of *phzA1-G1* and *phzA2-G2* in M18 genome with that in an outlier strain genome of *P. aeruginosa* PA7, were aligned using the *blastn* algorithm [35], revealing that the DNA sequence similarities were 79% and 84%, respectively. Using the RNA fold [36] web server, the base pairing probabilities were calculated and annotations with different colors as shown in Figure 4. The 5′-UTR folding free energy in the *phzA1-G1* gene cluster was determined to be −109.7 kcal/mol. Furthermore, in order to show the 5′-UTR dynamics, three suboptimal local structures from +230 to +337 nt were also predicted (Figure 4B). The free energies for these three structures were found to be very close, which would be expected to introduce only a small energy barrier between alternate structures. The above results indicated that the 5′-UTR from +230 to +337 nt of *phzA1-G1* may be a potential riboswitch and could change its structures easily to response to various environmental cues. The detailed mechanism(s) will be further investigated in future, by which the riboswitch variants in 5′-UTR of *phzA1-G1* control self-expression.

### Negative control of *phz* gene expression is achieved by GacA signal transduction via gene cluster *phzA2-G2*, rather than *phzA1-G1*

**Figure 5:**
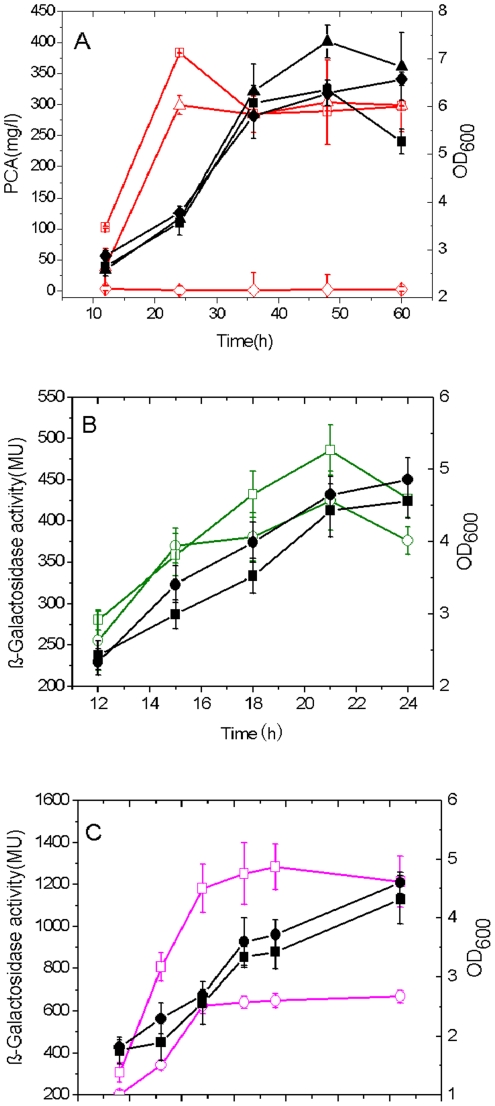
The PCA production was negatively regulated by GacA via *phzA2-G2* cluster mainly at post-transcriptional level. PCA production and cell growth curves in single *gacA* inactivated mutant M18ΔG (square), double *gacA* and *phzA1-G1* mutant M18ΔGΔP1 (triangle) or double *gacA* and *phzA2-G2* mutant M18ΔGΔP2 (diamond) (A). The cell growth and expression of transcriptional fusion pMP2C (B) or translational fusion pMP2L (C) in wild type strain M18 (circle) and mutant M18ΔG (square). Symbols: solid, growth curves; empty, PCA production or β-Galactosidase activities. All experiments were performed in triplicate, and each value is presented as the average ± standard deviation.

We have previously reported that PCA production was negatively controlled by the global regulator GacA [27], [34]. To extend our investigations, here, we sought to determine whether PCA repression occurred at both of the two *phz* gene clusters or only at one of them by exploiting the known GacA function. The double inactivated mutants of *gacA* and *phzA1-G1* or *phzA2-G2* gene cluster were constructed in wild type strain M18 and designated as M18ΔGΔP1 and M18ΔGΔP2. PCA production and cell growth were measured in the two double mutants and the single *gacA* mutant M18ΔG (Figure 5A). Contrast with growth curves obtained for the three strains revealed that different amounts of PCA were produced in each, and that PCA production was completely abrogated in the M18ΔGΔP2 mutant. Interestingly, an almost parallel PCA level of 300 mg/L accumulated in both mutants of M18ΔGΔP1 and M18ΔG, indicating that the negative control of *gacA* on PCA synthesis occurred in the *phzA2-G2* cluster, and not the *phzA1-G1* cluster.

To identify whether the negative control capability of GacA activity on the expression of *phzA2-G2* gene cluster occurred at the transcriptional or translational level, both the transcriptional fusion pMP2C and translational fusion pMP2L were delivered into the inactivated *gacA* mutant M18ΔG and the wild type strain M18. The transcriptional fusion level of the pMP2C was almost the same in the two mutants, while the translational fusion level of pMP2L in the *gacA* inactivated mutant M18ΔG was 2-fold higher than that in the wild type strain M18 (Figure 5B & 5C), indicating that the negative control of GacA activity on *phzA2-G2* expression occurred at the post-transcriptional level.

**Figure 6:**
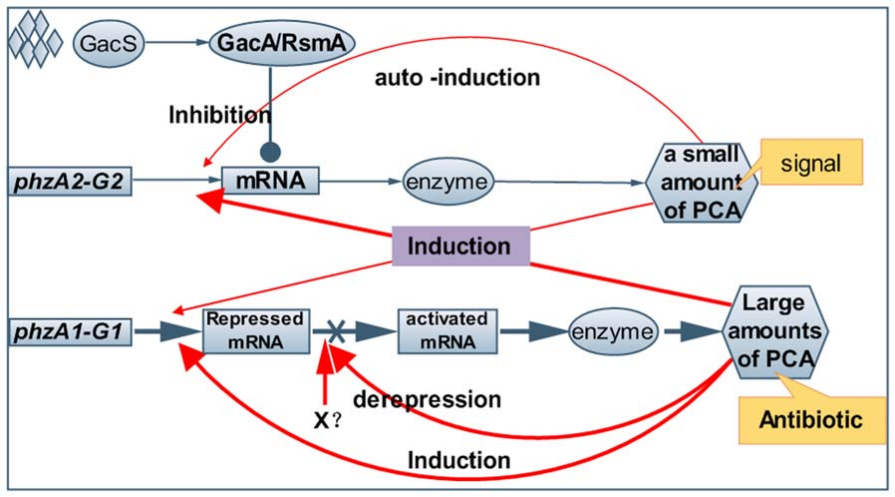
Integrative relationship of the Gac/Rsm signal transduction pathway and the expressions of two *phz* clusters. Diagrammatic representation of the integrative relationship of the Gac/Rsm signal transduction pathway and the expressions of two *phz* clusters in *Pseudomonas* sp. M18. Less efficiency of expression of *phzA2-G2* gene cluster produce a small amount of PCA signal molecule to auto-induce transcriptional activity itself and the expression of *phzA1-G1* gene cluster (red fine lines). The high efficiency expression of *phzA1-G1* at transcriptional level were blocked by its 5′-UTR region and could be relieved partial in post-transcriptional level by PCA or some unknown factor(s) (red thick lines). The interactions of induction and self-induced among two *phz* clusters resulted in a large amount of PCA production as antibiotics for bio-control. The *phzA2-G2* expression was negatively controlled mainly at the post-transcriptional level by regulator GacA in respond to environmental signals at overall level. Symbols: solid circle, inhibition; solid arrow, activation; diamond, environmental signals; X, unknown factor(s).

As summarized in Figure 6, our overall findings describe a correlation between the expressions of two *phz* gene clusters and the negative control on *phzA2-G2* expression by *gacA* activity in *Pseudomonas* sp. strain M18. It could be concluded that the concerted expressions of two *phz* gene clusters involves PCA induction and are regulated both at the transcriptional and post-transcriptional level by a feedback loop involving sequences in the 5′-UTR.

## Discussion

In this report, a correlation between the expression and regulation of two *phz* gene clusters involved in PCA synthesis was investigated in *Pseudomonas* sp. M18. Surprisingly, we identified a regulatory feedback loop of two *phz* gene clusters expression, which involved a small amount of PCA produced from the gene cluster *phzA2-G2* that could function as a signaling molecule to induce self-expression and to activate the expression of the *phzA1-G1* cluster, and could respond to adequate amounts of synthetic PCA in response to environmental cues. A hierarchical cascade pattern of the two LasIR and RhlIR quorum sensing (QS) systems is a well-documented cell-density dependent mechanism of intercellular communication in *P. aeruginosa* PAO1 that involves small diffusible signaling molecules in response to environmental changes [37]. In contrast to the various *N*-acyl-homoserine lactones (AHLs) that mediate the complicated QS systems, we found in *Pseudomonas* strain M18 that a single molecule, PCA, produced in different quantities by the two *phz* gene clusters acted as the functional mediator and was involved in the regulatory feedback loop of the expressions of two *phz* gene clusters.

Several distinguishing features were then characterized in this regulatory feedback loop. First, each of the two *phz* gene clusters plays distinctive role to produce different amounts of PCA. It has been previously reported in the literature that several antibiotics are able to function as molecular signals at lower sub-inhibitory concentrations and to enhance some potentially adaptive characteristics of the microbe to support its survival [38]–[40]. Phenazine has been demonstrated to play a dual role, both as a signaling molecule at a lower concentration and as an antibiotic at a relatively higher one [39], [40]. Notably, we found in this study that the two *phz* clusters play different roles to meet the requirements of producing different amounts of PCA molecules: the less efficiently expressed *phzA2-G2* gene cluster may produce a small amount of PCA which could then act as a signal to induce *phz* and many other genes expression, while the highly potentially expressed *phzA1-G1* gene cluster may be responsible for producing a larger amount of PCA which could then act as an antibiotics of warfare weapon to fight against fungal or bacterial phytopathogens in rhizosphere niches. The comparison of microarray-based transcriptome assay of wild type strain M18 and mutant M18ΔP2 showed that the small amounts of PCA produced from *phzA2-G2* could function as a signal molecule to activate the expressions of six other genes, including *mexGHI* (a general phenazine transporter), related genes beyond the gene cluster *phzA1-G1* and *phzA2-G2*, and inhibited expressions of 18 ORFs, including 11 of unknown function, and seven genes related to twitching motility (unpublished data).

Second, the feedback loop of the two *phz* gene clusters is regulated efficiently and flexibly to control the amounts of PCA produced presumably to prevent the potential toxic effect on cell growth. We found that the expression of *phzA1-G1* gene cluster has a potentially higher transcriptional ability, but the corresponding transcript was blocked by two *cis*-acting elements located, respectively, from about +1 to +90 nt and from +255 to +337 nt in the non-coding region of the *phzA1-G1* transcripts. We could assume that crucial on-off switches serve as a defensive countermeasure and tune the adaptive response in natural habits. Here, the secondary structure analysis of the non-coding elements downstream the transcriptional start site indicated that there might exist a potential riboswitch in the region between +230 and +337 nt. Riboswitches are complex folded RNA domains that directly bind a specific metabolite, such as an amino acid, and then control gene expression by exploiting changes in the RNA structure to influence transcription elongation or translation initiation [41], [42]. To date, all the riboswitches that have been discovered appear to function as repressors of gene expression in response to dynamic primary metabolites [41], [43]. Recent studies have made it clear that riboswitches represent a diverse and widespread form of regulation. Here, for the first time, we describe a predicted riboswitch located in +230 to +337 nt of 5′-UTR in strain M18 that may be involved in the regulation of *phz* gene expression, a region responsible for the biosynthesis of the secondary metabolite PCA compound related factor(s), although the detailed mechanism remains unknown. Further experiments should be performed to find more evidences to support the secondary structure predictions, such as cleavage assays in vitro transcription assays and in vitro analysis of the predicted RNA structures by crystallizing *phz* transcript (with or without PCA) and so on.

Third, we observed that the efficiency of PCA molecules corresponded to their functional ability as regulators to turn off the post-transcriptional repression; this capacity dropped to about 50% in mutant M18ΔP1, in comparison with that seen in M18ΔP2 (Figure 3B). It is likely that some unknown factor(s) could be involved in the inhibitory process; such factor(s) remain under investigation as we continue to detail the regulatory mechanism of the expressions of two *phz* gene clusters.

The highly conserved Gac/sRNA/Rsm system is a well-characterized signal transduction pathway and is a global regulatory mechanism in *Pseudomonas* spp. [44], [45]. The PCA derivative PYO is positively regulated by the Gac/sRNA/Rsm system, as has been demonstrated in *P. aeruginosa* PAO1 [33], [45]–[47]. However, we found that PCA production, the precursor event for PYO production, was negatively regulated by GacA activity, and was related to only the expression of *phzA2-G2* gene cluster, and not *phzA1-G1* expression in *Pseudomonas* sp. M18 at the post-transcriptional level. Furthermore, the secondary structure of the 5′-UTR in the *phzA2-G2* transcript was also predicted in this study (Figure 4A). The findings indicated the presence of a highly conserved loop structure which may act as a regulator involved in the stringent control of PCA biosynthesis by GacS/GacA signal transduction. Regardless, the higher structure complexity of the 5′-UTR in *phzA1-G1*, as compared with that of the *phzA2-G2*, suggested that there are key switches located in this non-coding region which may turn on or off gene expression in response to environment cues and which determines the amount of PCA synthesis. Whether this feature is typical to other *P. aeruginosa* isolated from different niches has yet to be determined.

What is clear from the current data is that the expressions of two *phz* gene clusters have developed specific interactive regulatory features under the evolutionary selective pressure imposed on *Pseudomonas* sp. M18. We suspect that the PCA molecule, other unknown factor(s), and RNA-mediated regulation are involved in the ability to turn on or off the expressions of two *phz* gene clusters, allowing for dominant and dynamic regulation of phenazine precursor PCA biosynthesis by using this regulatory feedback loop through 5′-UTRs in *Pseudomonas* sp. M18.

## Materials and Methods

### Bacterial strains and culture conditions

The bacterial strains and plasmids used in this study are listed in Table 2. *E. coli* were grown in Luria-Bertani (LB) medium. *Pseudomonas* sp. M18 and its derivatives were grown in King's medium B (KMB) containing peptone 20 g, glycerol 15 ml, K_2_HPO_4_ 0.392 g, MgSO_4_ 0.732 g per litre, and pigment-producing medium (PPM) with peptone 22 g, glucose 20 g, KNO_3_ 5 g per litre was used for PCA production. The antibiotics added in media were at the following concentrations (µg/ml): ampicillin (Ap) 100, spectimycin (Sp) 100, genamycin (Gm) 50, tetracycline (Tc) 120 for pseudomonads and Ap100 and Tc15 for *E. coli*. Routine incubation temperature was 37°C for *E. coli* and 28°C for *Pseudomonas* sp. M18 and its derivatives.

**Table 2:** Bacterial strains and plasmids used in this study.

Materials	Genotype, phenotype or characteristics	Reference or origin
Strains		
*E.coli* DH5α	*recA*1 *endA*1 *gyrA*96 *thi*1 *hsdR*17 (*rk^−^ mk^+^*) *supE*44 *re1A*1	[51]
SM10	*thr leu tonA lacy supE recA*::RP4-2-Tc::Mu Km^r^	[51]
*Pseudomonas* sp. M18	Wild type, PCA, Plt producer, Ap^r^ Sp^r^ Gm^s^ Km^s^	[26]
M18ΔP1	Δ*phzA1-G1*, *PhzA1*::Gm^r^	This study
M18ΔP2	Δ*phzA2-G2*, *PhzA2*::Gm^r^	This study
M18ΔG	Δ*gacA*, *gacA*::Km^r^,	[27]
M18ΔGΔP1	Δ*phzA1-G1*, *PhzA1*::Gm^r^ Δ*gacA*, *gacA*::Km^r^	This study
M18ΔGΔP2	Δ*phzA2-G2*, *PhzA2*::Gm^r^ Δ*gacA*, *gacA*::Km^r^	This study
Plasmids		
pBLS	pBluescript II KS+ cloning vector, ColE1 replicon, Ap^r^	This lab
pUCGM	Source of Gm^r^ cassette; Ap^r^, Gm^r^	Dieter Hass
pME18Tc	Gene replacement vector with multiple cloning sites from pUC18	[52]
pME6015	Pvs1-p15A *E. coli*-*Pseudomonas* shuttle vector for translational *lacZ* fusions and promoter probing, Tet^r^	Dieter Hass
pME6522	Pvs1-p15A *E. coli*-*Pseudomonas* shuttle vector for transcriptional *lacZ* fusions and promoter probing, Tet^r^	[47]
pMP1C	341 bp fragment from −340 to +1 upstream *phzA1-G1* transcription start site cloned into pME 6522	This study
pMP2C	345 bp fragment from −344 to +1 upstream *phzA2-G2* transcription start site cloned into pME 6522	This study
pMP1L	691 bp fragment from −340 to +351 upstream *phzA1-G1* translational start site cloned into pME 6015	This study
pMP2L	572 bp fragment from −344 to +228 upstream *phzA2-G2* translational start site cloned into pME 6015	This study
pMP1aC	430 bp fragment from −340 to +90 upstream *phzA1-G1* transcription start site cloned into pME 6522	This study
pMP1bC	595 bp fragment from −340 to +255 upstream *phzA1-G1* transcription start site cloned into pME 6522	This study
pMP1cC	677 bp fragment from −340 to +337 upstream *phzA1-G1* transcription start site cloned into pME 6522	This study

### DNA manipulation and cloning procedures

Restriction endonucleases, *Taq*, LA-*Taq* and *Pfu* DNA polymerase, DNA molecular mass markers, and other associated products, were used as recommended by the respective manufacturer (TaKaRa; MBI; Fermentas). Plasmid DNA was prepared on a small-scale using the MiniBEST plasmid purification kit, version 2.0 (TaKaRa). Genomic DNA was extracted and purified from *Pseudomonas* sp. M18 using an EZ spin column genomic DNA isolation kit (Bio Basiv, Inc). Restriction enzyme digestions, ligations and agarose gel electrophoresis were performed using standard methods. Restriction fragments were purified from agarose gels by DNA gel extraction kit (Axgen). The chemically-synthesized oligonucleotide primers used in this study were listed in Table 3.

**Table 3:** Primers designed for this study.

Oligonucleotide name	Sequence (5′ - 3′) and restriction site (enzyme)
P1GSP1	GTTTCCCTGTACCGCTGA
P2GSP1	CGGTAAACCCTTTCAACC
P1GSP2	TCGTCGCTCGGTAAGG
P2GSP2	GCGAATCTCCGCCAGT
AAP	GGCCACGCGTCGACTAGTACGGGIIGGGIIGGGIIG
P1U	AATATTGAGCTCTGCTCGCCTTCATCGC (*Sac*I)
P1D	TATATACTGCAGCCCTTCGGCAGGAGATA (*Pst*I)
P2U	AATATTGAGCTC CTACCTTCGGCGACCTG (*Sac*I)
P2D	CATATACTGCAGGATCGTCCATAGTTCACCC (*Pst*I)
P1CU	CTGATAGAATTCCACATTTCCGTAACCCGA (*Eco*RI)
P1CD	CTACAGCTGCAGGGATTGCATAAAACACAGA (*Pst*I)
P2CU	CTGAGCGAATTCTGGCCAGATAGCGTTTG (*Eco*RI)
P2CD	TCAGATCTGCAGGGGACAAACTCATAGACGC (*Pst*I)
P1LU	CTGATAGAATTCCACATTTCCGTAACCCGA (*Eco*RI)
P1LD	TATACTGCAGTTCCCTGTACCGCTGACC (*Pst*I)
P2LU	GCTGTAGAATTCCTCAACTCCAGCAACAAGG (*Eco*RI)
P2LD	TAGCTACTGCAGCCCTTTCAACCGTTGGTA (*Pst*I)
P1aCD	ATATAGCTGCAGTCGTTAAGGTGCGACAGA (*Pst*I)
P1bCD	TATACACTGCAG TTCGTCGCTCGGTAAG (*Pst*I)
P1cCD	TATATACTGCAGGCGCCGCCTCCGAGAGGG (*Pst*I)
PCU	GTATCCTCAAGGGCTATGC
PCD	GGGATGAACCGAGATAGAC
PA1U	TCAGCGGTACAGGGAAAC
PA1D	TCCGTGGTCCAGTTGC
PA2U	GGTTGAAAGGGTTTACCG
PA2D	TCCGTGGTCCAGTTGC
Rpod1^a^	GAGCGGGAGGAGCGTTTAC
Rpod2^a^	CGGGCAAAAAATAAGCAGAGG

a These oligonucleotides were from [28].

### Mapping the transcriptional start site

Rapid amplification of 5′-cDNA element (5′-RACE) was performed to identify the transcriptional start sites for the two *phz* clusters by using Invitrogen's 5′-RACE system. Briefly, total RNA was isolated at the early exponential phase (OD_600_ = 1.5) from *Pseudomonas* sp. M18 cultures using the Nucleospin RNAII kit (Macherey-Nagel) and treated with RQ1 RNase-free DNase (Promega). Gene specific primers P1GSP1 and P2GSP1 were designed and first strand cDNA were synthesized using RevertAid™ first strand cDNA synthesis kit (Fermentas); a homopolymeric tail was then added to the 3′-cDNA using terminal deoxynucleotidyl transferase (TdT) and dCTP. PCR was accomplished using nested gene-specific primers P1GSP2 and P2GSP2, a novel deoxyinosine-containing abridged anchor primer (AAP), and the poly(C) tailed cDNA as template. The 5′-RACE products were analyzed, purified and placed into individual pMD18T vectors (TaKaRa) to determine the transcriptional start sites of the two *phz* clusters by direct sequencing, respectively.

### Construction of two *phz* gene cluster-inactivated mutants, M18ΔP1 and M18ΔP2

Two primer pairs, P1U-P1D and P2U-P2D (Table 3), were designed according to the nucleotide sequences located between the coding regions of *phzA1* and *phzB1* or *phzA2* and *phzB2* in the two *phz* gene clusters in *Pseudomonas* sp. M18. A 2.2 kb and a 1.7 kb DNA fragment was PCR amplified from strain M18 genomic DNA and confirmed by direct sequencing. After *Sac*I-*Pst*I digestion, the fragments were cloned into pEX18, a conjugatable counter-selective suicide plasmid to obtain recombinant plasmid pEX18P1 and pEX18P2 in *E. coli* SM10.

A 0.8 kb *Sma*I fragment containing the gentamycin (Gm) resistance cassette from pUCGm was inserted into the *Sma*I trimmed site of *phzA1* and *phzA2* gene for both plasmids pEX18P1 and pEX18P2, resulting in the recombinant constructs pEX18P1Gm and pEX18P2Gm, respectively, in *E. coli* SM10; the constructs were ultimately mobilized into *Pseudomonas* sp. M18 by biparental mating. The clones in which a double crossover had occurred were selected on plates containing Gm50, Sp100 and 5% (w/v) sucrose. The *phzA1-G1* and *phzA2-G2* chromosomally inactivated insertion mutants of M18ΔP1 and M18ΔP2 were selected for Gm-resistant (Gm^r^) colonies followed by Tc-sensitive (Tc^s^) screening and confirmed by PCR and sequencing.

### Construction of *phz1‘-’lacZ* and *phz2‘-’lacZ* transcriptional and translational fusions

All the primers used in the construction were listed in the Table 3. A 341 bp PCR fragment from −340 to +1 (relative to the transcriptional start point) containing the *phzA1-G1* gene cluster promoter region and a 345 bp PCR fragment from −344 to +1 containing the *phzA2-G2* promoter region was amplified respectively from strain M18 genomic DNA using two primer pairs, P1CU-P1CD and P2CU-P2CD. The products were then cloned into *Eco*RI-*Pst*I digested pME6522 respectively to generate two transcriptional fusions of pMP1C and pMP2C. A 691 bp PCR fragment (from −340 to +351 which contained the promoter region and partially encompassed the *phzA1* gene) and a 572 bp PCR fragment (from −344 to +228 which contained the promoter region along with a portion of the *phzA2* gene) were amplified using two primer pairs, P1LU-P1LD and P2LU-P2LD. The products were then cloned into *Eco*RI-*Pst*I digested pME6015 to generate two translational fusions of pMP1L and pMP2L.

Using the primer pairs P1CU-P1aCD, P1CU-P1bCD and P1CU-P1cCD, three fragments of 430 bp, 595 bp and 677 bp (from −340 to +90, +255 and +337, respectively) were PCR amplified and cloned into pME6522 to generate transcriptional fusion pMP1aC, pMP1bC and pMP1cC. All PCR fragments were amplified from strain M18 genome DNA and confirmed by direct sequencing.

### Quantitative reverse transcriptase (QRT-) PCR

*Pseudomonas* sp. M18 and its derivatives, M18ΔP1 and M18ΔP2, were grown in PPM medium to an OD_600_ of 2.0–2.5 (exponential phase) and 3.5–4.0 (stationary phase). Total RNA was extracted from the cell pellet using a Nucleospin RNAII kit (MN), according to the manufacturers' instructions and including the optional DNase treatment step. The extracted RNA was used as template for cDNA generation, which was next applied to a random primed reverse transcriptase reaction (Fermentas, MBI) following the manufacturer's protocol and then used as template for quantitative PCR (Rotor-Gene 6000; Corbett Life Sciences) by means of the SYBR Green I detection system. The signal was standardized to *rpoD* (a house keeping gene) using the following equation: relative expression = 2^−Δ(ΔCTsample-ΔCTstandard)^, where CT (cycle threshold) was determined automatically by the Rotor-Gene 6000 PCR software. Primers for QRT-PCR were designed using Primer5 software. Criteria for primer design were: a melting temperature of 58°C, primer length of 20 nt, and an amplified PCR product of about 200 bp. Samples were assayed in triplicate.

Two primer pairs, PA1U-A1D and PA2U-PA2D, were designed and based on distinctive sequence in the *phzA1* and *phzA2* genes in *Pseudomonas* sp. M18. The total transcriptional level of the two *phz* gene clusters was determined using the primer PCU-PCD designed from *phzC*, the *phzA1* and *phzA2* gene transcription level as representatives of the *phzA1-G1* and *phzA2-G2* transcripts, respectively, in *Pseudomonas* sp. M18. The constitutively expressed gene *rpoD* was used as the internal control to verify the absence of significant variation in cDNA levels for all samples. RealMasterMix (SYBR Green I; Tiangen, China) was used to carry out real-time PCR. PCRs were run with following program: one step of 1 min at 94°C, 40 cycles of 94°C for 15 s, 58°C for 20 s, and 68°C for 30 s. PCR analyses for each strain were repeated three times. The amount of target gene *phzC*, *phzA1* and *phzA2*, normalized to the level of the reference *rpoD* and calibrated relative to the lowest of them, was calculated as a 2^−ΔΔCT^.

### Supplementation of exogenous PCA in culture

After 12 h culture, during the exponential phase (OD_600_ = 2.0∼2.5), purified PCA compound (20 mM dissolved in ethanol) was added to attain a final concentration of 0.1 mM; the analytical pure ethanol solvent was used as control. Following PCA addition, the culture was further incubated for approximately 60 h until the decline phase (OD_600_ = 5.5∼6.5).

### Assays for PCA and β-Galactosidase

Extraction and quantification of PCA from the culture suspension were performed using the described methods [48]. β-Galactosidase assays were carried out according to the method of Miller [49]. All experiments were performed in triplicate.

### Secondary structure analysis of 5′- untranslated region (UTRs) of two *phz* gene clusters

The 5′-UTRs of *phzA1-G1* and *phzA2-G2* were aligned with the outlier *P. aeruginosa* strain PA7 genome using the *blastn* algorithm [35]. Then, the conserved RNA secondary structures were predicted using the RNA fold [36] web server (http://rna.tbi.univie.ac.at/cgi-bin/RNAfold.cgi), and the base pairing probabilities were calculated. Subsequently, portions of the 5′-UTR of *phzA1-G1* were taken to predict three suboptimal structures, and the free energies of optimal and suboptimal structures were calculated with mfold [50].
